# Polyamines, cardiorespiratory fitness, body composition, and mitochondrial function in older patients with coronary artery disease

**DOI:** 10.1093/ehjopen/oeag150

**Published:** 2026-09-21

**Authors:** Christian Thorup, Thomas Hvid Jensen, Chris Nørgaard Agerbo Jeppesen, Andreas Bugge Tinggaard, Mogens Johannsen, Jakob Hansen, Christian Lodberg Hvas, Charlotte Lock Rud, Bent Roni Ranghøj Nielsen, Jakob Wang, Jean Farup, Niels Jessen, Henrik Wiggers

**Affiliations:** Department of Cardiology, Aarhus University Hospital, Palle Juul-Jensens Boulevard 69, Aarhus 8200, Denmark; Department of Clinical Medicine, Aarhus University, Palle Juul-Jensens Boulevard 99, Aarhus 8200, Denmark; Department of Cardiology, Aarhus University Hospital, Palle Juul-Jensens Boulevard 69, Aarhus 8200, Denmark; Department of Clinical Medicine, Aarhus University, Palle Juul-Jensens Boulevard 99, Aarhus 8200, Denmark; Department of Cardiology, Aarhus University Hospital, Palle Juul-Jensens Boulevard 69, Aarhus 8200, Denmark; Department of Clinical Medicine, Aarhus University, Palle Juul-Jensens Boulevard 99, Aarhus 8200, Denmark; Department of Cardiology, Aarhus University Hospital, Palle Juul-Jensens Boulevard 69, Aarhus 8200, Denmark; Department of Clinical Medicine, Aarhus University, Palle Juul-Jensens Boulevard 99, Aarhus 8200, Denmark; Department of Forensic Medicine, Aarhus University, Palle Juul-Jensens Boulevard 99, Aarhus 8200, Denmark; Department of Forensic Medicine, Aarhus University, Palle Juul-Jensens Boulevard 99, Aarhus 8200, Denmark; Department of Clinical Medicine, Aarhus University, Palle Juul-Jensens Boulevard 99, Aarhus 8200, Denmark; Department of Hepatology and Gastroenterology, Aarhus University Hospital, Palle Juul-Jensens Boulevard 99, Aarhus 8200, Denmark; Department of Clinical Medicine, Aarhus University, Palle Juul-Jensens Boulevard 99, Aarhus 8200, Denmark; Department of Hepatology and Gastroenterology, Aarhus University Hospital, Palle Juul-Jensens Boulevard 99, Aarhus 8200, Denmark; Department of Cardiology, Aarhus University Hospital, Palle Juul-Jensens Boulevard 69, Aarhus 8200, Denmark; Department of Clinical Medicine, Aarhus University, Palle Juul-Jensens Boulevard 99, Aarhus 8200, Denmark; Steno Diabetes Center Aarhus, Aarhus University Hospital, Palle Juul-Jensens Boulevard 11, Aarhus, Denmark; Department of Biomedicine, Aarhus University, Høegh-Guldbergs Gade 10, Aarhus 8000, Denmark; Steno Diabetes Center Aarhus, Aarhus University Hospital, Palle Juul-Jensens Boulevard 11, Aarhus, Denmark; Department of Biomedicine, Aarhus University, Høegh-Guldbergs Gade 10, Aarhus 8000, Denmark; Steno Diabetes Center Aarhus, Aarhus University Hospital, Palle Juul-Jensens Boulevard 11, Aarhus, Denmark; Department of Biomedicine, Aarhus University, Høegh-Guldbergs Gade 10, Aarhus 8000, Denmark; Department of Cardiology, Aarhus University Hospital, Palle Juul-Jensens Boulevard 69, Aarhus 8200, Denmark; Department of Clinical Medicine, Aarhus University, Palle Juul-Jensens Boulevard 99, Aarhus 8200, Denmark

**Keywords:** Coronary artery disease, Ageing, Spermidine, Spermine, Cardiorespiratory fitness, Mitochondrial function

## Abstract

**Aims:**

Higher dietary spermidine intake has been associated with lower cardiovascular mortality. Spermidine and spermine support cellular homeostasis and mitochondrial function across species, but their relevance to physical and mitochondrial function in humans remains unclear. We examined associations of dietary spermidine and circulating and skeletal muscle spermidine and spermine with physical performance, body composition, and mitochondrial respiratory capacity in older patients with coronary artery disease.

**Methods and results:**

We included 187 patients with coronary artery disease [median age 72 (68–76) years; 80% men; 95% with prior coronary revascularization]. The primary outcomes were peak oxygen uptake and appendicular lean mass. Each doubling of dietary spermidine intake was associated with higher peak oxygen uptake (β 2.6 mL·kg^−1^·min^−1^, 95% CI 0.9–4.4; *P* = 0.003). This association remained after adjustment for age, sex, and physical activity. Dietary spermidine intake was also associated with higher appendicular lean mass, but this association was attenuated after adjustment. Among secondary outcomes, higher spermidine intake was associated with higher appendicular lean mass/body mass index and lower body fat percentage. In a subgroup, higher spermidine intake was associated with greater mitochondrial respiration. In exploratory analyses, a higher skeletal muscle spermine-to-spermidine ratio showed similar associations (all *P* ≤ 0.035).

**Conclusion:**

These observational findings warrant randomized trials to determine whether dietary spermidine interventions may influence physical and mitochondrial function in older patients with coronary artery disease.

## Graphical Abstract

**Graphical Abstract:**
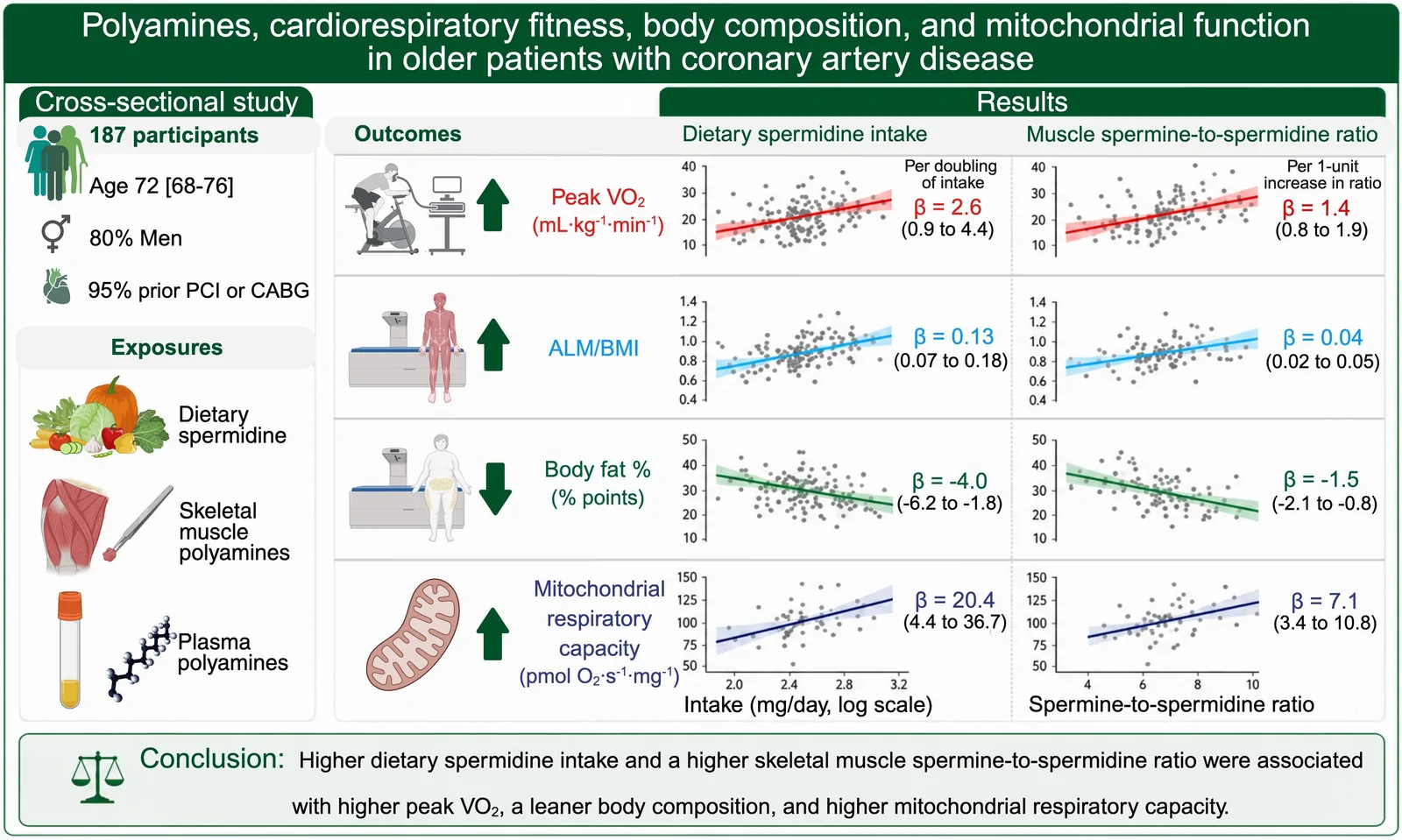

## Introduction

As populations age, strategies to preserve physical function and independence are increasingly important for healthy ageing. This challenge is particularly relevant in older patients with coronary artery disease (CAD), in whom mitochondrial dysfunction may contribute to reduced cardiorespiratory fitness and impaired physical function.^1,2^ Polyamine metabolism has gained attention as a potential pathway for preserving cardiovascular health, skeletal muscle function, and mitochondrial homeostasis during ageing.^3–14^

Spermidine and spermine are naturally occurring polyamines found in all forms of life and are essential for cellular growth and homeostasis.^8,15–18^ Spermidine is derived from dietary intake, endogenous synthesis, and production by the gut microbiota.^19,20^ Dietary spermidine is abundant in plant-derived foods, and higher intake has been associated with lower cardiovascular and all-cause mortality.^21,22^ Moreover, endogenous polyamine synthesis declines with age,^4,23,24^ and plasma spermidine concentrations may be lower in patients with CAD.^25^ Preclinical studies suggest that spermidine exerts cardioprotective effects, enhances mitochondrial function, and extends lifespan.^3,4,6–8,10,26^ However, evidence linking spermidine to physical and mitochondrial function in humans remains limited.

We hypothesized that dietary spermidine intake and plasma and skeletal muscle polyamine concentrations would be positively associated with cardiorespiratory fitness, skeletal muscle mass, and mitochondrial function in older patients with CAD. We therefore investigated the associations of dietary spermidine intake, spermine and spermidine plasma and skeletal muscle concentrations with peak oxygen uptake (peak VO_2_), appendicular lean mass (the combined lean mass of the arms and legs; ALM), and skeletal muscle mitochondrial respiratory capacity. We additionally explored associations with the spermine-to-spermidine ratio because lower whole-blood ratios have previously been reported in individuals with low skeletal muscle mass.^27^

## Methods

### Study design

This cross-sectional study was reported in accordance with the Strengthening the Reporting of Observational Studies in Epidemiology (STROBE) guidelines. Ethical approval was obtained from the Central Denmark Region Committee on Health Research Ethics, and all participants provided written informed consent (approval no. 1-10-72-71-23). The study was conducted at Aarhus University Hospital, Denmark.

### Study participants

Participants were included between November 2023 and March 2025 at their baseline visit of the POLYCAD trial (ClinicalTrials.gov identifier NCT06186102).^18^ Eligible participants were aged ≥65 years and had established chronic CAD, defined by previous myocardial infarction and/or coronary revascularization. All participants were required to have a left ventricular ejection fraction (LVEF) > 40% on screening echocardiography. To enrol a population at increased cardiometabolic risk, participants were additionally required to meet at least two of the following criteria: known hypertension; known type 2 diabetes; body mass index ≥ 30 kg/m^2^ at screening; a previously documented LVEF < 40% with subsequent improvement to >40% at screening; left atrial volume index ≥ 30 mL/m^2^; or left ventricular wall thickness ≥ 1.1 cm on screening echocardiography. These criteria were selected to enrich the cohort for cardiometabolic and structural cardiac abnormalities associated with increased risk of functional decline and heart failure.

Key exclusion criteria included unstable coronary syndrome, significant valvular heart disease judged by the investigator to affect study outcomes or participant safety, severe peripheral artery disease, permanent atrial fibrillation, pacemaker therapy, chronic kidney disease or chronic liver disease, active malignancy within five years before screening, and inability to provide informed consent. Use of dual antiplatelet therapy or anticoagulant therapy precluded skeletal muscle biopsy but not participation in the study.^18^

### Food frequency questionnaire

Dietary spermidine and spermine intake was assessed using a targeted 159-item food frequency questionnaire (FFQ). It was reviewed by experts including two investigators (C.L.R. and C.L.H.), pilot tested, and evaluated against 3-day weighed dietary records. All interviews were conducted by the same registered dietitian. A more detailed description has been reported previously.^28^

### Blood sampling and muscle biopsy

Venous blood samples and skeletal muscle biopsies were collected between 08:00 and 10:00 a.m. after a ≥10-h overnight fast. Muscle biopsies were obtained from the vastus lateralis of the non-dominant leg using the percutaneous Bergström technique with manual suction.^29^ Samples for respirometry were processed immediately, whereas samples for liquid chromatography-tandem mass spectrometry (LC-MS/MS) analysis were snap-frozen in liquid nitrogen and stored at −80°C. Blood samples were collected in EDTA tubes, processed within 30 min, and centrifuged at 2000 × *g* for 10 min at 4°C, and plasma was stored at −80°C until analysis.

### Quantification of spermidine and spermine by liquid chromatography-tandem mass spectrometry

Spermidine and spermine in plasma and skeletal muscle were quantified by targeted LC-MS/MS using stable isotope-labelled internal standards. Polyamines were extracted from plasma and skeletal muscle tissue samples and analysed by ultra-performance liquid chromatography coupled to triple-quadrupole mass spectrometry with positive electrospray ionization and multiple reaction monitoring mode. Quantification was based on linear calibration curves, and method validation demonstrated acceptable precision and trueness. The analytical method has been described previously.^28^

### Physical performance

Cardiopulmonary exercise testing (CPET) was performed on a cycle ergometer (JAEGER Vyntus CPX, Vyaire Medical, Mettawa, IL) using a standardized ramp protocol to symptom-limited exhaustion, targeting an exercise duration of 8–12 min. Blood pressure and electrocardiography were continuously monitored. Gas exchange was measured continuously during exercise to obtain oxygen consumption, carbon dioxide production, and respiratory exchange ratio. Peak VO_2_ was defined as the highest 15-s average and normalized to body mass. A respiratory exchange ratio ≥ 1 at peak exercise was used to evaluate whether peak effort was obtained.

Maximal unilateral isometric knee-extension strength was assessed using a dynamometer (CON-TREX; Physiomed, Schnaittach, Germany), and the highest peak torque from three voluntary contractions of the dominant leg was used for analysis. Handgrip strength was measured using a handheld dynamometer (Charder Medical, Taichung City, Taiwan), and the highest value from three attempts with the dominant hand was used for analysis.

Submaximal physical performance was assessed using the 30-s sit-to-stand test and a 6-min walk test according to guidelines.

Physical activity was assessed using an accelerometer (Axivity AX3, Newcastle upon Tyne, UK), and average acceleration and step count was derived. Participants were instructed to wear an accelerometer on the right wrist for 14 days, and analyses included those with ≥8 days of ≥16 h of daily wear. Accelerometry procedures have been described previously.^30^

### Body composition

Body composition, including ALM, total lean mass, fat mass, and body fat percentage, was assessed by dual-energy X-ray absorptiometry (DXA) (Hologic, Marlborough, MA, USA) after an overnight fast of at least 10 h. Appendicular lean mass was divided by BMI to derive the appendicular lean mass-to-body mass index ratio (ALM/BMI). This measure was selected because it accounts partly for body size and adiposity and may identify clinically relevant low muscle mass more effectively than absolute ALM in patients with cardiovascular disease and heart failure.^31^ All scans were performed and analysed by trained personnel according to standardized procedures.

### High-resolution respirometry

Mitochondrial respiration in permeabilized skeletal muscle fibres was assessed by high-resolution respirometry (Oxygraph-2k; Oroboros, Innsbruck, Austria) using a substrate-uncoupler-inhibitor titration protocol for non-fatty acid-linked respiration as described previously.^32^ Mitochondrial respiratory indices of complex I, complex II, and maximal electron transport capacity were measured. Mitochondrial respiratory indices were normalized to citrate synthase activity in sensitivity analyses to account for variation in mitochondrial content and assess respiratory capacity relative to mitochondrial abundance.

### Statistical analysis

The predefined primary outcomes were peak VO_2_ and ALM.^18^ Continuous variables are presented as mean (SD) or median (IQR), as appropriate. Associations were analysed using linear regression models, with skewed variables natural log-transformed. Primary associations were assessed using unadjusted linear regression models. To evaluate robustness to potential confounding, sensitivity analyses were adjusted for age and sex and additionally for accelerometer-derived physical activity. No adjustment was made for multiple comparisons. Analyses were performed using complete cases for the variables included in each model, and missing data were not imputed. Consequently, sample sizes varied across outcomes and were smaller in fully adjusted models requiring accelerometer-derived physical activity data. Model assumptions were evaluated by visual inspection of quantile-quantile plots of the residuals. Regression coefficients (β) are reported with 95% confidence intervals (CI) and two-sided *P* values. Dietary spermidine intake was natural log-transformed before analysis because of its right-skewed distribution. For interpretability, effects are expressed per doubling of dietary spermidine intake by β × ln(2). Pearson correlation coefficients were reported in Supplementary material online, *Table S1*. Data were collected in Research Electronic Data Capture (REDCap) hosted at Aarhus University, Denmark. Statistical analyses were performed in R version 4.4.2 (R Foundation for Statistical Computing, Vienna, Austria) and RStudio (Posit Software, PBC, Boston, MA, USA).

## Results

### Patient characteristics

A total of 187 participants were included from the baseline assessment of the POLYCAD trial. The participant flow is shown in *Figure 1*, and baseline characteristics are presented in *Tables 1* and *2*. The median age was 72 years (IQR, 68–76), the mean BMI was 28 (SD, 4) kg/m^2^, and 149 participants (80%) were men. A history of acute myocardial infarction was present in 99 participants (53%), and 178 (95%) had undergone percutaneous coronary intervention, coronary artery bypass grafting, or both. Hypertension and type 2 diabetes were present in 174 (93%) and 49 (26%) participants, respectively.

**Figure 1:**
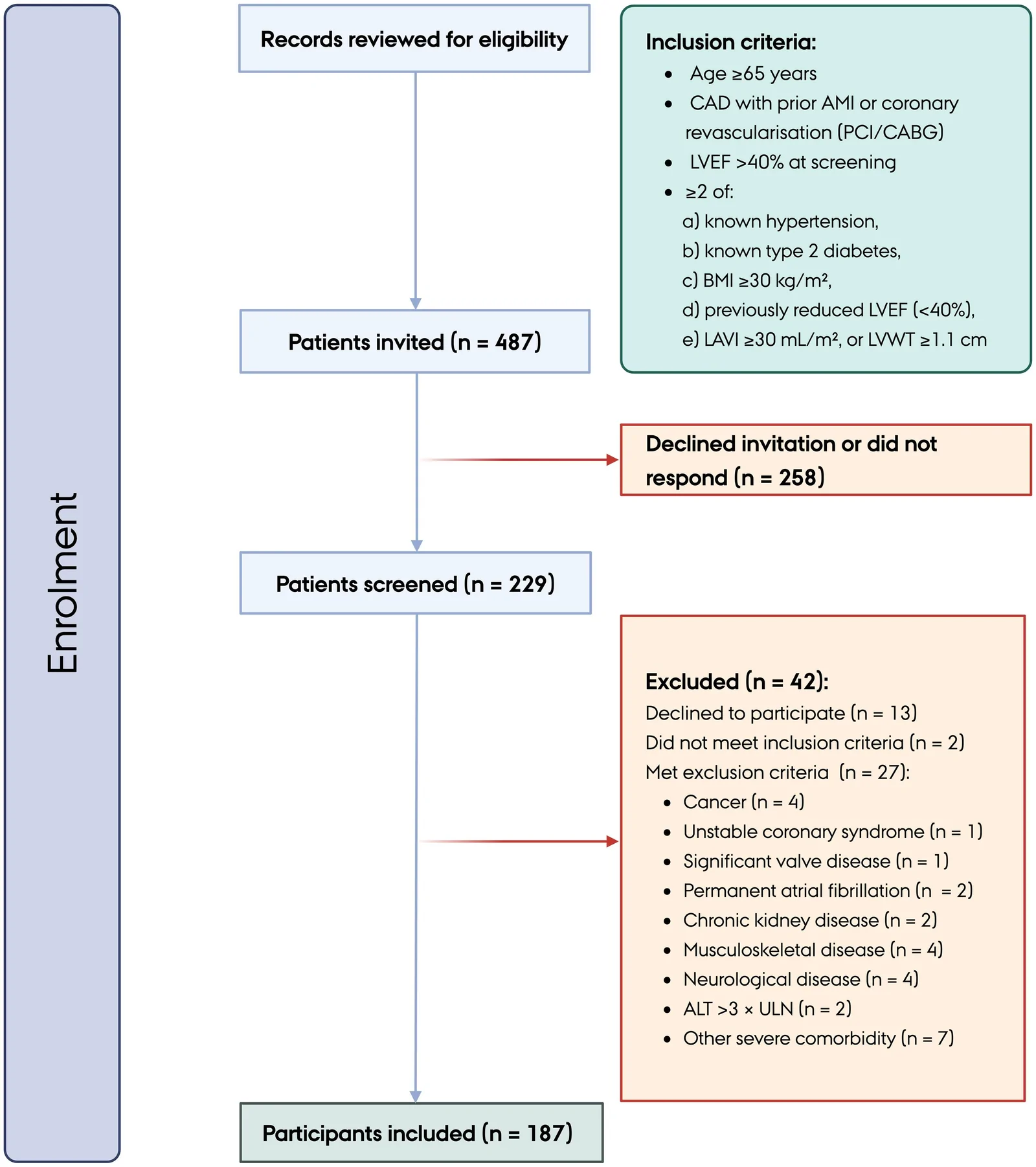
Flow diagram of participant enrolment (*n* = 187). ALT, alanine aminotransferase; BMI, body mass index; LAVI, left atrial volume index; LVEF, left ventricular ejection fraction; LVWT, left ventricular wall thickness; ULN, upper limit of normal.

**Table 1:** Baseline characteristics of the study population

Characteristic	Overall (*n* = 187)	Women (*n* = 38)	Men (*n* = 149)
Age, years, median (IQR)	72 (68 to 76)	72 (68 to 77)	72 (68 to 75)
LVEF, %, median (IQR)	60 (55 to 60)	60 (55 to 60)	60 (53 to 60)
Medical history, *n* (%)			
Prior AMI	99 (53)	20 (53)	79 (53)
STEMI	51 (27)	11 (29)	40 (27)
NSTEMI	51 (27)	10 (26)	41 (28)
Prior PCI	153 (82)	27 (71)	126 (85)
Prior CABG	40 (21)	9 (24)	31 (21)
Hypertension	174 (93)	35 (93)	139 (93)
Type 2 diabetes	49 (26)	8 (21)	41 (28)
Atrial fibrillation/flutter	28 (15)	3 (8)	25 (17)
Prior stroke	8 (4)	4 (11)	4 (3)
COPD	9 (5)	3 (8)	6 (4)
NYHA functional class, *n* (%)			
Class I	144 (77)	26 (68)	118 (79)
Class II	43 (23)	12 (32)	31 (21)
CCS angina grade, *n* (%)			
Grade 0	151 (81)	30 (79)	121 (81)
Grade I	34 (18)	6 (16)	28 (19)
Grade II	2 (1)	2 (5)	0 (0)
Educational attainment, *n* (%)			
Primary school	15 (8)	4 (11)	11 (7)
Upper secondary	72 (39)	19 (50)	53 (36)
Vocational education	28 (15)	4 (11)	24 (16)
Bachelor’s degree	44 (24)	10 (26)	34 (23)
Master’s degree or higher	28 (15)	1 (3)	27 (18)
Medications, *n* (%)			
Beta-blockers	85 (45)	16 (42)	69 (46)
ACE inhibitors	50 (27)	6 (16)	44 (30)
ARBs	95 (51)	22 (58)	73 (49)
MRAs	13 (7)	2 (5)	11 (7)
SGLT2 inhibitors	29 (16)	3 (8)	26 (17)
Loop diuretics	7 (4)	0 (0)	7 (5)
Thiazide diuretics	45 (24)	8 (21)	37 (25)
DOACs	29 (16)	4 (11)	25 (17)
Antiplatelet therapy	157 (84)	34 (89)	123 (83)
Calcium antagonists	95 (51)	18 (47)	77 (52)
Nitrates	40 (21)	7 (18)	33 (22)
Lipid-lowering drugs	183 (98)	35 (92)	148 (99)
Metformin	37 (20)	7 (18)	30 (20)
GLP-1 receptor agonists	20 (11)	3 (8)	17 (11)
Insulin	7 (4)	0 (0)	7 (5)

Data are presented as median (IQR) or *n* (%). Participants may have had more than one type of prior myocardial infarction.

ACE, angiotensin-converting enzyme; AMI, acute myocardial infarction; ARB, angiotensin II receptor blocker; CABG, coronary artery bypass grafting; CCS, Canadian Cardiovascular Society; COPD, chronic obstructive pulmonary disease; DOAC, direct oral anticoagulant; GLP-1, glucagon-like peptide-1; LVEF, left ventricular ejection fraction; MRA, mineralocorticoid receptor antagonist; NSTEMI, non-ST-segment elevation myocardial infarction; NYHA, New York Heart Association; PCI, percutaneous coronary intervention; SGLT2, sodium-glucose cotransporter-2; STEMI, ST-segment elevation myocardial infarction.

**Table 2:** Baseline polyamine measures and clinical assessments

Measure	Overall	Women	Men
Polyamine measures			
Spermidine intake, mg/day, *n* = 180, median, (IQR)	11.5 (9.5 to 14.3)	10.3 (8.1 to 11.9)	11.7 (9.8 to 14.6)
Spermine intake, mg/day, *n* = 180, median (IQR)	7.8 (6.4 to 9.5)	6.0 (4.9 to 7.0)	8.2 (7.0 to 9.8)
Plasma spermidine, ng/mL, *n* = 185, median (IQR)	21.0 (16.2 to 27.1)	20.4 (17.4 to 26.7)	21.4 (16.0 to 27.4)
Plasma spermine, ng/mL, *n* = 185, median (IQR)	15.1 (11.6 to 19.0)	15.5 (13.1 to 19.0)	15.1 (11.5 to 19.0)
Skeletal muscle spermidine, ng/g, *n* = 101, median (IQR)	7120 (6200 to 8500)	9110 (7400 to 10 400)	7010 (5900 to 7700)
Skeletal muscle spermine, ng/g, *n* = 101, median (IQR)	48 000 (43 600 to 53 300)	46 900 (43 200 to 49 600)	48 200 (43 700 to 53 600)
Physical performance (*n* = 185)			
Peak VO_2_, mL·kg^−1^·min^−1^, mean (SD)	20.5 (4.9)	18.4 (4.4)	21.1 (4.9)
Peak workload, W, mean (SD)	143.9 (46.1)	98.8 (31.9)	155.6 (41.8)
6-min walk distance, m, mean (SD)	519.8 (114.0)	492.9 (132.0)	526.8 (109.0)
Sit-to-stand performance, repetitions, mean (SD)	16.4 (7.8)	15.3 (5.4)	16.7 (8.3)
Handgrip strength, kg, mean (SD)	36.8 (9.3)	23.9 (5.5)	40.2 (6.7)
Knee extensor strength, Nm, mean (SD)	127.8 (44.5)	84.1 (22.4)	139.0 (41.8)
Knee flexor strength, Nm, mean (SD)	119.1 (44.8)	75.4 (22.0)	130.2 (42.3)
Body composition (*n* = 187)			
Body weight, kg, mean (SD)	86.3 (16.0)	70.7 (14.0)	90.3 (14.0)
BMI, kg/m^2^, mean (SD)	28.3 (4.0)	25.8 (5.0)	28.9 (4.0)
Lean mass, kg, mean (SD)	56.6 (10.5)	41.9 (5.8)	60.4 (7.9)
Appendicular lean mass, kg, mean (SD)	23.6 (4.9)	16.9 (2.6)	25.4 (3.8)
ALM/height2, kg/m^2^, mean (SD)	7.7 (1.2)	6.2 (0.9)	8.1 (1.0)
ALM/BMI, mean (SD)	0.8 (0.2)	0.7 (0.1)	0.9 (0.1)
Total fat mass, kg, mean (SD)	28.2 (8.6)	27.2 (9.9)	28.4 (8.3)
Body fat, %, mean (SD)	32.2 (6.4)	38.2 (6.1)	30.6 (5.5)
Laboratory measurements (*n* = 185)			
HDL cholesterol, mmol/L, median (IQR)	1.2 (1.0 to 1.5)	1.4 (1.3 to 1.7)	1.2 (1.0 to 1.4)
LDL cholesterol, mmol/L, median (IQR)	1.4 (1.2 to 1.7)	1.5 (1.3 to 1.9)	1.4 (1.1 to 1.6)
HbA1c, mmol/mol, median (IQR)	40 (38 to 46)	40 (38 to 43)	40 (38 to 46)
Creatinine, µmol/L, median (IQR)	79 (70 to 92)	67 (58 to 74)	84 (74 to 96)
hs-CRP, mg/L, median (IQR)	0.7 (0.3 to 1.4)	0.6 (0.3 to 0.9)	0.7 (0.4 to 1.4)
Mitochondrial respiratory capacity (*n* = 51)			
Complex I + II OXPHOS, pmol O_2_·s^−1^·mg^−1^, mean (SD)	94.8 (23.8)	75.0 (17.5)	97.9 (23.3)
Complex I OXPHOS, pmol O_2_·s^−1^·mg^−1^, mean (SD)	57.5 (12.9)	48.7 (10.5)	58.9 (12.8)
ETS capacity, pmol O_2_·s^−1^·mg^−1^, mean (SD)	119.0 (30.8)	88.0 (22.7)	124.0 (29.2)
Physical activity (*n* = 175)			
Average acceleration, milligravity units, mean (SD)	21.8 (5.7)	24.3 (5.9)	21.1 (5.5)
Step count, steps/day, mean (SD)	7911 (3083)	9231 (3062)	7704 (3023)

Data are presented as mean (SD) or median (IQR).

ALM, appendicular lean mass; BMI, body mass index; ETS, electron transport system; HbA1c, glycated haemoglobin; HDL, high-density lipoprotein; hs-CRP, high-sensitivity C-reactive protein; LDL, low-density lipoprotein; OXPHOS, oxidative phosphorylation; peak VO_2_, peak oxygen uptake.

### Spermidine and spermine levels

A total of 180 participants (96%) completed the FFQ. Median dietary spermidine intake was 11.5 mg/day (IQR, 9.5–14.3), and median spermine intake was 7.8 mg/day (IQR, 6.4–9.5). Plasma polyamine concentrations were available for 185 patients (99%). Median plasma concentrations were 21.0 (IQR, 16.2–27.1) ng/mL for spermidine and 15.1 (IQR, 11.6–19.0) ng/mL for spermine. Skeletal muscle biopsies were obtained from 101 participants (54%). Median skeletal muscle concentrations were 7120 (IQR, 6200–8500) ng/g for spermidine and 48 000 (IQR, 43 600–53 300) ng/g for spermine (*Table 2*).

### Dietary spermidine intake and physical performance

A total of 177 participants completed both the FFQ and CPET (*Table 3*). The mean (SD) peak VO_2_ was 20.5 (5) mL·kg^−1^·min^−1^. Higher dietary spermidine intake was associated with higher peak VO_2_ (2.6 mL·kg^−1^·min^−1^ per doubling of intake; 95% CI, 0.9–4.4; *P* = 0.003), a higher peak workload (27.6 W per doubling of intake; 95% CI, 11.6–43.5; *P* < 0.001), and better 30-s sit-to-stand performance (4.3 repetitions per doubling of intake; 95% CI, 1.5–7.0; *P* = 0.003). These associations remained after adjustment for age, sex, and accelerometer-derived physical activity.

**Table 3:** Associations of dietary spermidine intake and skeletal muscle spermine-to-spermidine ratio with selected study outcomes

Exposure	Outcome	*n*	Crude β (95% CI)	Crude *P*	Adjusted β (95% CI)	Adjusted *P*
Dietary spermidine intake (per doubling in mg/day)						
	Peak VO_2_, mL·kg^−1^·min^−1^	177	2.6 (0.9 to 4.4)	0.003	2.3 (0.7 to 3.9)	0.004
	Peak workload, W	177	27.6 (11.6 to 43.5)	<0.001	21.9 (9.4 to 34.5)	<0.001
	ALM/BMI	180	0.13 (0.07 to 0.18)	<0.001	0.09 (0.04 to 0.13)	<0.001
	Body fat, %	180	−4.0 (−6.2 to −1.8)	<0.001	−2.5 (−4.4 to −0.7)	0.008
	Mitochondrial complex I OXPHOS, pmol O_2_·s^−1^·mg^−1^	51	10.3 (1.5 to 18.7)	0.022	8.5 (0.1 to 17.0)	0.048
	Mitochondrial complex I + II OXPHOS, pmol O_2_·s^−1^·mg^−1^	51	20.4 (4.4 to 36.7)	0.014	18.4 (3.0 to 33.8)	0.020
Skeletal muscle spermine-to-spermidine ratio (per 1 unit increase in the ratio)						
	Peak VO_2_, mL·kg^−1^·min^−1^	101	1.37 (0.81 to 1.90)	<0.001	0.94 (0.3 to 1.5)	0.003
	Peak workload, W	101	12.8 (7.6 to 18.0)	<0.001	5.82 (0.8 to 10.8)	0.023
	ALM/BMI	101	0.04 (0.02 to 0.05)	<0.001	0.02 (0.003 to 0.04)	0.017
	Body fat, %	101	−1.5 (−2.1 to −0.8)	<0.001	−1.0 (−1.6 to −0.34)	0.003
	Mitochondrial complex I OXPHOS, pmol O_2_·s^−1^·mg^−1^	51	3.7 (1.7 to 5.8)	<0.001	2.4 (0.2 to 4.6)	0.035
	Mitochondrial complex I + II OXPHOS, pmol O_2_·s^−1^·mg^−1^	51	7.1 (3.4 to 10.8)	<0.001	4.7 (0.5 to 8.8)	0.027
	ETS capacity, pmol O_2_·s^−1^·mg^−1^	51	10.5 (5.9 to 15.0)	<0.001	8.12 (3.0 to 13.2)	0.003

Crude β coefficients (95% confidence intervals) and *P* values are from unadjusted linear regression models. Adjusted β coefficients (95% confidence intervals) and *P* values are multivariable models adjusted for age, sex, and physical activity measured as average acceleration in milligravity units. For interpretation, β coefficients and 95% confidence intervals for natural log-transformed spermidine intake were multiplied by ln(2) and therefore represent the difference in the outcome per doubling of intake. The complete set of unadjusted, age- and sex-adjusted, fully adjusted, and Pearson correlation analyses is presented as Supplementary material online, *Table S1*.

ALM, appendicular lean mass; CI, confidence interval; ETS, electron transport system; OXPHOS, oxidative phosphorylation; peak VO_2_, peak oxygen uptake.

In unadjusted models, higher dietary spermidine intake was also associated with higher handgrip strength (4.2 kg per doubling of intake; 95% CI, 1.0–7.6; *P* = 0.01), but not 6-min walk distance or knee-extension strength.

### Dietary spermidine intake and body composition

A total of 180 participants completed both the FFQ and DXA (*Table 3*). The mean (SD) ALM was 23.6 (5) kg. Higher dietary spermidine intake was associated with higher ALM/BMI (0.13 per doubling of intake; 95% CI, 0.07–0.18; *P* < 0.001) and inversely associated with total fat mass (−3.7 kg per doubling of intake; 95% CI, −6.6 to −0.7; *P* = 0.015) and body fat percentage (−4 percentage points per doubling of intake; 95% CI, −6.2 to −1.8; *P* < 0.001). The associations remained after adjusting for age, sex, and physical activity.

Higher dietary spermidine intake was associated with ALM (2.1 kg per doubling of intake; 95% CI, 0.4–3.8; *P* = 0.014) in an unadjusted model.

### Dietary spermidine intake and mitochondrial respirometry

Skeletal muscle mitochondrial respiration was assessed in 51 patients (*Tables 2* and *3*). The respirometry subgroup was descriptively similar to the overall cohort in terms of age, BMI, cardiometabolic history, peak VO_2_, body composition, and dietary spermidine intake (see Supplementary material online, *Table S2*). In the respirometry group, mean (SD) complex I capacity was 57.5 (12.9) pmol O_2_·s^−1^·mg^−1^, combined complex I + II capacity was 94.8 (23.8) pmol O_2_·s^−1^·mg^−1^, and electron transport system capacity was 119 (30.8) pmol O_2_·s^−1^·mg^−1^. Higher dietary spermidine intake was associated with greater complex I-linked (10.3 pmol O_2_·s^−1^·mg^−1^ per doubling of intake; 95% CI 1.5–18.7; *P* = 0.022) and complex I + II-linked respiration (20.4 pmol O_2_·s^−1^·mg^−1^ per doubling of intake; 95% CI 4.4–36.7; *P* = 0.014). These associations remained after adjustment for age, sex, and accelerometer-derived physical activity and normalization to citrate synthase activity.

### Dietary spermidine intake and blood biomarkers

In 181 participants, higher dietary spermidine intake was associated with lower plasma triglycerides (−0.25 mmol/L per doubling of intake; 95% CI, −0.46 to −0.05; *P* = 0.016) and higher HDL cholesterol (0.14 mmol/L per doubling of intake; 95% CI, 0.02–0.25; *P* = 0.022), in unadjusted models.

### Plasma and skeletal muscle spermidine and spermine

In 185 participants, plasma spermidine and spermine concentrations were not associated with any outcomes of interest. In 101 participants, skeletal muscle spermidine concentrations were inversely associated with peak VO_2_, muscle strength, and lean mass and positively associated with body fat percentage. These associations were attenuated after adjustments for age and sex. No associations were found between skeletal muscle spermine concentrations and the outcomes.

### Skeletal muscle spermine-to-spermidine ratio and physical performance

In an exploratory analysis, we investigated the associations of the spermine-to-spermidine ratio and physical performance. A higher skeletal muscle spermine-to-spermidine ratio was associated with higher peak VO_2_ (β = 1.37 mL·kg^−1^·min^−1^; 95% CI, 0.81–1.90; *P* < 0.001) and higher peak workload (β = 12.8 W; 95% CI, 7.6–17.9; *P* < 0.001). These associations remained after adjustment for age, sex, and accelerometer-derived physical activity (*Table 3*). The ratio was also associated with higher 6-min walk distance and handgrip strength, but these associations did not remain after adjustment.

### Skeletal muscle spermine-to-spermidine ratio and body composition

A higher skeletal muscle spermine-to-spermidine ratio was associated with higher ALM/BMI (β = 0.04; 95% CI, 0.02–0.05; *P* < 0.001), lower total fat mass (β = −1.3 kg; 95% CI, −2.2 to −0.3; *P* = 0.013), and lower body fat percentage (β = −1.5 percentage points; 95% CI, −2.1 to −0.8; *P* < 0.001) (*Table 3*).

### Skeletal muscle spermine-to-spermidine ratio and mitochondrial respiration

A higher skeletal muscle spermine-to-spermidine ratio was associated with greater complex I-linked respiratory capacity (β = 3.73 pmol O_2_·s^−1^·mg^−1^; 95% CI, 1.71 to 5.75; *P* < 0.001), complex I + II-linked respiratory capacity (β = 7.06 pmol O_2_·s^−1^·mg^−1^; 95% CI, 3.36 to 10.75; *P* < 0.001), and maximal electron transport system capacity (β = 10.49 pmol O_2_·s^−1^·mg^−1^; 95% CI, 5.93 to 15.04; *P* < 0.001) (*Table 3*). Associations remained statistically significant after normalization to citrate synthase activity: complex I capacity (β = 5.65, 95% CI 1.50 to 9.80; *P* = 0.009), complex I + II capacity (β = 10.80, 95% CI 3.33 to 18.28; *P* = 0.006), and maximal electron transport system capacity (β = 15.55, 95% CI 7.26 to 23.85; *P* < 0.001).

### Skeletal muscle spermine-to-spermidine ratio and cardiometabolic blood biomarkers

A higher spermine-to-spermidine ratio was associated with higher HDL cholesterol (β = 0.06 mmol/L; 95% CI, 0.02–0.10; *P* < 0.01) and lower hs-CRP (β = −0.78 mg/L; 95% CI, −1.43 to −0.12; *P* = 0.02) and remained after adjustment for age, sex, and accelerometer-derived physical activity.

## Discussion

We examined associations of dietary, circulating, and skeletal muscle polyamine measures with physical performance, body composition, and skeletal muscle mitochondrial respiratory capacity in patients with CAD. In the primary unadjusted analyses, higher dietary spermidine intake was associated with higher peak VO_2_ and greater ALM. The association with peak VO_2_ remained after adjustment for age, sex, and physical activity, whereas the association with absolute ALM was attenuated. Higher dietary spermidine intake was also associated with higher ALM/BMI, lower body fat percentage, and greater skeletal muscle mitochondrial respiratory capacity, and these associations remained after adjustments. In exploratory analyses, a higher skeletal muscle spermine-to-spermidine ratio was also associated with higher peak VO_2_ and ALM/BMI, lower body fat percentage, and greater skeletal muscle mitochondrial respiratory capacity.

### Spermidine intake

In the population-based Bruneck cohort of 829 participants followed for 20 years, higher dietary spermidine intake was associated with lower cardiovascular and all-cause mortality.^21^ Higher dietary spermidine intake was also associated with lower risk of fatal or clinically overt heart failure and fewer major cardiovascular events.^4^ These ﬁndings are further supported by larger studies using data from NHANES and UK Biobank.^22,33^ Dietary spermidine intake has also been associated with lower BMI and waist circumference, as well as lower fasting glucose, fasting insulin, HOMA-IR, HbA1c, and lower incidence of diabetes.^34,35^

Our findings extend previous observational evidence linking dietary spermidine intake to cardiometabolic health and longevity by demonstrating associations with objectively measured cardiorespiratory fitness, comprehensive DXA-derived body composition, and directly measured skeletal muscle mitochondrial respiratory capacity in a well-characterised cohort of older patients with CAD. These associations persisted after adjustment for age, sex, and accelerometer-measured physical activity assessed over 14 days, suggesting that they were not explained solely by a more active lifestyle. Nevertheless, reverse causation and residual dietary confounding remain possible. Dietary spermidine may be a marker of an overall healthy dietary pattern rather than the factor responsible for the observed associations.

Preclinical studies indicate that dietary spermidine is absorbed from the small intestine, enters the circulation, and is subsequently distributed to peripheral tissues, including skeletal muscle.^36,37^ We previously reported that higher spermidine intake was modestly associated with higher plasma spermidine concentration, but not with skeletal muscle spermidine concentrations.^28^ Previous interventional studies have reported only modest effects of spermidine supplementation on circulating polyamine concentrations and mainly spermine.^38,39^ These observations provide limited support for a substantial effect of higher dietary intake on circulating or skeletal muscle polyamine concentrations. They also do not support the use of plasma or skeletal muscle polyamine concentrations as biomarkers of habitual spermidine intake. However, most clinical studies have used doses below 10% of average daily intake, and dose and duration required to achieve clinically relevant effects remain unknown.^40–42^

### Skeletal muscle spermidine and spermine

Higher skeletal muscle spermidine concentrations were associated with lower peak VO_2_ and ALM in unadjusted analyses. However, these associations were substantially attenuated and no longer statistically significant after adjustment for age and sex. As we previously reported, women had significantly higher skeletal muscle spermidine concentrations than men.^28^ These findings suggest that the unadjusted inverse associations may have been driven partly by sex-related differences. Sex differences in polyamine concentrations may be partly explained by hormonal regulation of polyamine metabolism.^43,44^ In rats, females have shown a higher spermidine/*N*^1^-acetyltransferase-to-polyamine oxidase activity ratio and a higher spermidine-to-spermine ratio than males.^45^ However, the mechanisms underlying sex differences in polyamine metabolism remain insufficiently studied in humans.

We previously reported that skeletal muscle spermine concentrations were approximately seven-fold higher than spermidine concentrations.^28^ Spermine has been suggested to serve as a storage reservoir for maintaining adequate levels of free spermidine.^46^ However, both spermidine and spermine seem to have essential biological functions. Spermidine is the more extensively studied of the polyamines, and dietary spermidine has been consistently linked to autophagy and mitochondrial function in preclinical studies.^3–8^ Spermidine and polyamine-pathway regulation have been implicated in skeletal muscle stem cell (satellite cell) function, muscle fibre integrity, mitochondrial quality control, and protection against atrophy or myopathic pathology.^3,5,9–14^ In addition, dietary spermidine supplementation in mice protects against obesity and induces dose-dependent reductions in fat mass and adipocyte size.^34,47,48^ Spermine has been less extensively studied than spermidine but appears to play important roles in redox regulation and DNA stabilization.^46,49^

The skeletal muscle spermine-to-spermidine ratio was positively associated with mitochondrial respiratory capacity. Mitochondrial indices remained associated after normalization to citrate synthase activity, suggesting that the association may also reflect greater respiratory capacity relative to mitochondrial abundance. The spermine-to-spermidine ratio was also associated with ALM/BMI, an indicator of skeletal muscle mass relative to BMI. ALM/BMI has previously been positively associated with physical function in patients with heart failure.^31^ Our finding is consistent with a previous study reporting lower spermine-to-spermidine ratios in patients with sarcopenia than in non-sarcopenic controls.^27^ These findings suggest that the relative balance between spermine and spermidine may be linked to skeletal muscle phenotype and mitochondrial respiratory capacity, but the underlying biological mechanisms remain uncertain. Spermidine-dependent hypusination of eukaryotic initiation factor 5A has been implicated in cellular homeostasis and mitochondrial function, but the spermine-to-spermidine ratio is not a direct measure of polyamine flux, enzymatic activity, or spermidine utilization, and its physiological significance in human skeletal muscle requires further investigation.^6,7,26^

### Limitations

This cross-sectional design precludes inferences about causality and directionality, and findings should be interpreted cautiously. The predefined exposures were dietary spermidine intake and plasma and skeletal muscle spermidine concentrations; the predefined primary outcomes were peak VO_2_ and ALM. All other analyses, including those involving the skeletal muscle spermine-to-spermidine ratio and mitochondrial respiratory capacity, were exploratory. Because multiple secondary and exploratory associations were examined without formal adjustment for multiplicity, the possibility of type I error cannot be excluded. Dietary spermidine intake was estimated using a targeted 159-item FFQ administered by a registered dietitian and evaluated against 3-day weighed dietary records. Nevertheless, FFQ-derived estimates may be affected by recall and reporting errors and by variation in the polyamine content of foods arising from differences in production, storage, and preparation. If non-differential, such measurement error may attenuate the observed associations towards the null, although systematic reporting differences and bias in either direction cannot be excluded. Although habitual dietary patterns are generally reasonably stable, dietary intake may change over time; therefore, a single FFQ may not fully reflect long-term intake. We measured polyamines in plasma and skeletal muscle. However, their associations with dietary intake were too weak to be considered reliable biomarkers of intake. Further work to identify and validate biomarkers of habitual spermidine intake would be valuable. Despite sensitivity analyses adjusting for age, sex, and accelerometer-measured physical activity, residual confounding cannot be excluded. In particular, higher spermidine intake may reflect a generally healthier dietary pattern or lifestyle, and other socioeconomic factors, comorbidity burden, and medication use may also have influenced the observed associations. Skeletal muscle biopsies obtained from the thigh may not reflect whole-body skeletal muscle metabolism, and biopsy and respirometry data were available only for subsets of participants. Although participants with biopsy and respirometry data were descriptively similar to the overall cohort for measured characteristics, selection bias cannot be excluded. In particular, anticoagulant therapy or dual antiplatelet therapy precluded muscle biopsy and may have selected a clinically more stable subgroup. The muscle and mitochondrial findings may therefore not be fully generalizable to patients with a greater thrombotic or comorbidity burden. Finally, the cohort comprised older patients with CAD, which limits generalizability to other populations.

## Conclusions

Higher dietary spermidine intake and a higher skeletal muscle spermine-to-spermidine ratio were associated with higher peak VO_2_, greater ALM/BMI, lower body fat percentage, and greater skeletal muscle mitochondrial respiratory capacity in older patients with CAD. These observational findings suggest that dietary spermidine intake and skeletal muscle polyamine metabolism may be relevant to cardiorespiratory fitness, body composition, and mitochondrial function, but do not establish causality. Further mechanistic studies and randomized trials are warranted to determine whether increasing spermidine intake or targeting polyamine metabolism can preserve physical capacity and improve clinical outcomes.

## Supplementary Material

oeag150_Supplementary_Data

## Data Availability

De-identified participant data, a data dictionary, and analytical code may be made available upon reasonable request to the corresponding author, subject to institutional approvals, ethical requirements, and applicable data protection regulations.
