# Supplementary material for: Polyamines, cardiorespiratory fitness, body composition, and mitochondrial function in older patients with coronary artery disease
Source: Eur Heart J Open. 2026 Sep 21;6(5):oeag150. doi: 10.1093/ehjopen/oeag150 (PMC13649080; doi:10.1093/ehjopen/oeag150)
Supplement: Supplementary file 1 [file oeag150_supplementary_data.docx]

**Supplementary material**

**Supplementary Table 1 Dietary spermidine intake, skeletal muscle spermine-to-spermidine ratio and study outcomes**

| **Outcome** | **Crude β (95% CI)** | **Crude P** | **Age- and sex-adjusted P** | **Fully adjusted P** | **Pearson r** |
| --- | --- | --- | --- | --- | --- |
| **Dietary spermidine intake, log scale** | | | | | |
| **Physical performance, n = 177** |  |  |  |  |  |
| Peak VO₂, mL·kg⁻¹·min⁻¹ | 3.8 (1.3 to 6.3) | 0.003 | 0.008 | 0.004 | 0.22 |
| Peak workload, W | 39.8 (16.7 to 62.8) | <0.001 | 0.002 | <0.001 | 0.25 |
| Sit-to-stand, repetitions | 6.2 (2.2 to 10.1) | 0.003 | 0.003 | 0.008 | 0.23 |
| 6-min walk distance, m | 43 (-17 to 103) | 0.16 | 0.17 | 0.21 | 0.11 |
| Handgrip strength, kg | 6.1 (1.4 to 10.9) | 0.01 | 0.09 | 0.16 | 0.19 |
| Knee extensor strength, Nm | 17.2 (-5.5 to 40.0) | 0.14 | 0.41 | 0.30 | 0.11 |
| **Body composition, n = 180** |  |  |  |  |  |
| ALM/BMI | 0.18 (0.10 to 0.26) | <0.001 | <0.001 | <0.001 | 0.33 |
| Appendicular lean mass, kg | 3.1 (0.6 to 5.5) | 0.01 | 0.10 | 0.11 | 0.18 |
| Total lean mass, kg | 4.6 (-0.6 to 9.9) | 0.08 | 0.54 | 0.53 | 0.13 |
| Body fat, % | -5.8 (-8.9 to -2.6) | <0.001 | 0.005 | 0.008 | -0.26 |
| Total fat mass, kg | -5.3 (-9.5 to -1.0) | 0.02 | 0.02 | 0.03 | -0.18 |
| BMI, kg/m² | -2.4 (-4.6 to -0.2) | 0.03 | 0.007 | 0.008 | -0.16 |
| **Mitochondrial respiratory capacity, n = 51** |  |  |  |  |  |
| Complex I OXPHOS, pmol O₂·s⁻¹·mg⁻¹ | 14.9 (2.2 to 27.0) | 0.02 | 0.02 | 0.04 | 0.32 |
| Complex I+II OXPHOS, pmol O₂·s⁻¹·mg⁻¹ | 29.5 (6.3 to 53.0) | 0.01 | 0.01 | 0.02 | 0.34 |
| ETS capacity, pmol O₂·s⁻¹·mg⁻¹ | 20.6 (-11.0 to 52.0) | 0.20 | 0.22 | 0.27 | 0.18 |
| **Skeletal muscle spermine-to-spermidine ratio** | | | | | |
| **Physical performance, n = 101** |  |  |  |  |  |
| Peak VO₂, mL·kg⁻¹·min⁻¹ | 1.37 (0.81 to 1.90) | <0.001 | <0.001 | 0.003 | 0.40 |
| Peak workload, W | 12.8 (7.6 to 18.0) | <0.001 | 0.003 | 0.02 | 0.44 |
| Sit-to-stand, repetitions | 1.0 (-0.07 to 2.1) | 0.07 | 0.16 | 0.31 | 0.19 |
| 6-min walk distance, m | 14.6 (0.13 to 29.1) | 0.05 | 0.36 | 0.95 | 0.20 |
| Handgrip strength, kg | 1.1 (0.05 to 2.2) | 0.04 | 0.10 | 0.11 | 0.21 |
| Knee extensor strength, Nm | 5.7 (0.83 to 10.5) | 0.02 | 0.94 | 0.76 | 0.23 |
| **Body composition, n = 101** |  |  |  |  |  |
| ALM/BMI | 0.04 (0.02 to 0.05) | <0.001 | 0.02 | 0.02 | 0.39 |
| Appendicular lean mass, kg | 0.7 (0.2 to 1.3) | 0.01 | 0.68 | 0.64 | 0.25 |
| Total lean mass, kg | 1.3 (0.1 to 2.6) | 0.04 | 0.21 | 0.22 | 0.21 |
| Body fat, % | -1.5 (-2.1 to -0.8) | <0.001 | 0.003 | 0.003 | -0.40 |
| Total fat mass, kg | -1.3 (-2.2 to -0.3) | 0.01 | 0.001 | 0.05 | -0.25 |
| BMI, kg/m² | -0.38 (-0.90 to 0.16) | 0.17 | 0.002 | 0.07 | -0.14 |
| **Mitochondrial respiratory capacity, n = 51** |  |  |  |  |  |
| Complex I OXPHOS, pmol O₂·s⁻¹·mg⁻¹ | 3.7 (1.7 to 5.8) | <0.001 | 0.003 | 0.03 | 0.46 |
| Complex I+II OXPHOS, pmol O₂·s⁻¹·mg⁻¹ | 7.1 (3.4 to 10.8) | <0.001 | 0.003 | 0.03 | 0.48 |
| ETS capacity, pmol O₂·s⁻¹·mg⁻¹ | 10.5 (5.9 to 15.0) | <0.001 | <0.001 | 0.003 | 0.55 |

**Supplementary Table 1** Values are β coefficients (95% confidence intervals) from unadjusted linear regression models. Dietary spermidine intake was natural log-transformed; estimates are presented per unit increase in log-transformed intake. For the skeletal muscle spermine-to-spermidine ratio, estimates represent the difference in outcome per 1-unit increase in the ratio on the original scale. Age- and sex-adjusted models included age and sex. Fully adjusted models additionally included physical activity measured as average acceleration in milligravity units. Abbreviations: ALM, appendicular lean mass; BMI, body mass index; CI, confidence interval; ETS, electron transport system; OXPHOS, oxidative phosphorylation; peak VO₂, peak oxygen uptake.

**Supplementary Table 2 Characteristics of the overall cohort and mitochondrial respirometry subgroup**

| **Characteristic** | **Overall cohort (n = 187)** | **Muscle biopsy group (n = 101)** | **Respirometry group (n = 51)** |
| --- | --- | --- | --- |
| Age, years, median (IQR) | 72 (68 to 76) | 72 (69 to 76) | 72 (70 to 74) |
| Men, n (%) | 149 (80) | 83 (81) | 44 (86) |
| **Clinical characteristics** |  |  |  |
| BMI, kg/m², mean (SD) | 28.3 (4.0) | 28.3 (4.6) | 28.0 (4.3) |
| Type 2 diabetes, n (%) | 49 (26) | 30 (29) | 13 (25) |
| Prior AMI, n (%) | 99 (53) | 59 (58) | 29 (57) |
| Prior PCI and/or CABG, n (%) | 178 (95) | 98 (96) | 48 (94) |
| **Functional and dietary characteristics** |  |  |  |
| Spermidine intake, mg/day, median (IQR) | 11.5 (9.5 to 14.3) | 10.9 (9.5 to 13.6) | 11.4 (10.1 to 13.6) |
| Peak VO₂, mL·kg⁻¹·min⁻¹, mean (SD) | 20.5 (4.9) | 20.5 (5.3) | 21.1 (5.0) |
| ALM/BMI, mean (SD) | 0.80 (0.20) | 0.83 (0.16) | 0.83 (0.17) |

**Supplementary Table 2** Data are presented as mean (SD), median (IQR), or n (%). The muscle biopsy group and the respirometry subgroup are included in the overall cohort; therefore, no between-group statistical tests were performed. Abbreviations: ALM, appendicular lean mass; AMI, acute myocardial infarction; BMI, body mass index; CABG, coronary artery bypass grafting; PCI, percutaneous coronary intervention; VO₂, oxygen uptake.
